# Photovoltaic Cell Surface Defect Detection via Subtle Defect Enhancement and Background Suppression

**DOI:** 10.3390/mi16091003

**Published:** 2025-08-30

**Authors:** Yange Sun, Guangxu Huang, Chenglong Xu, Huaping Guo, Yan Feng

**Affiliations:** School of Computer and Information Technology, Xinyang Normal University, Xinyang 464000, China; yangesun@xynu.edu.cn (Y.S.); gxhuang@xynu.edu.cn (G.H.); clxu@xynu.edu.cn (C.X.); hpguo@xynu.edu.cn (H.G.)

**Keywords:** photovoltaic cell defects detection, attention mechanism, subtle defect detection, deep learning methods

## Abstract

As the core component of photovoltaic (PV) power generation systems, PV cells are susceptible to subtle surface defects, including thick lines, cracks, and finger interruptions, primarily caused by stress and material brittleness during the manufacturing process. These defects substantially degrade energy conversion efficiency by inducing both optical and electrical losses, yet existing detection methods struggle to precisely identify and localize them. In addition, the complexity of background noise and other factors further increases the challenge of detecting these subtle defects. To address these challenges, we propose a novel PV Cell Surface Defect Detector (PSDD) that extracts subtle defects both within the backbone network and during feature fusion. In particular, we propose a plug-and-play Subtle Feature Refinement Module (SFRM) that integrates into the backbone to enhance fine-grained feature representation by rearranging local spatial features to the channel dimension, mitigating the loss of detail caused by downsampling. SFRM further employs a general attention mechanism to adaptively enhance key channels associated with subtle defects, improving the representation of fine defect features. In addition, we propose a Background Noise Suppression Block (BNSB) as a key component of the feature aggregation stage, which employs a dual-path strategy to fuse multiscale features, reducing background interference and improving defect saliency. Specifically, the first path uses a Background-Aware Module (BAM) to adaptively suppress noise and emphasize relevant features, while the second path adopts a residual structure to retain the original input features and prevent the loss of critical details. Experiments show that PSDD outperforms other methods, achieving the highest $mAP_{50}$ scores of 93.6% on the PVEL-AD.

## 1. Introduction

Solar energy, as a clean and renewable resource, has attracted worldwide attention in the global pursuit of carbon neutrality and the mitigation of climate change [1]. Photovoltaic cells, as the core devices for converting solar energy into electricity, have their manufacturing quality directly determining energy conversion efficiency and decisively influencing the overall system performance [2]. However, during the manufacturing process of PV cells, various defects may arise on the cell surface due to internal stress concentration, uneven thermal treatment, or minor damage [3]. These defects not only reduce energy conversion efficiency by creating carrier recombination centers [4], but also trigger localized hotspot effects, further accelerating the degradation of cell performance [5]. Therefore, the accurate and timely detection of surface defects in PV cells is of critical importance for the sustainable development of the photovoltaic industry.

In recent years, researchers have continuously explored the application of deep learning in detecting surface defects in PV cells. Akram et al. [6] proposed a simplified CNN architecture and incorporated data augmentation to improve defect detection performance while maintaining low model complexity; however, its limited representational capacity results in suboptimal performance in capturing complex defect features. Subsequently, Wang et al. [7] developed YOLO-PV, a customized network architecture specifically designed for EL images, which emphasizes fine-grained feature fusion to balance detection accuracy and computational efficiency; however, its generalization capability across different defect types remains limited. More recently, Liu et al. [8] proposed a CNN-based method that combines contrast-limited adaptive histogram equalization with a global context attention mechanism to enhance the recognition of fine-grained defects in EL images, although its relatively complex network structure also results in higher training and inference costs.

**Figure 1:**
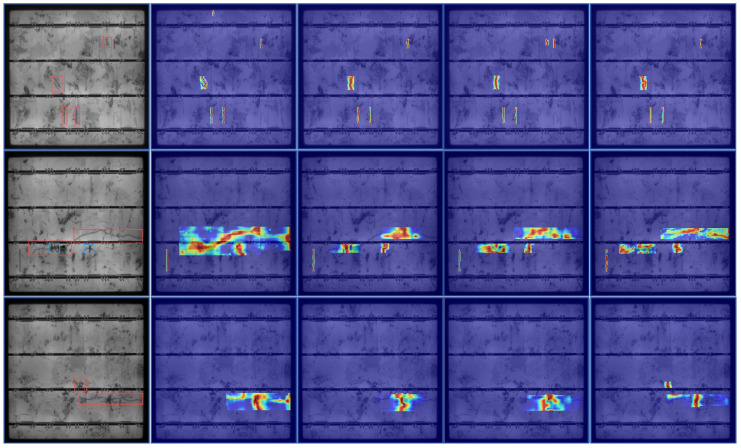
Heatmaps generated by our method and three object detection methods. The red boxes indicate crack defects, while the blue boxes represent star-shaped crack defects.

Existing deep learning methods for PV cell defect detection have demonstrated promising results; however, under complex background noise, achieving precise detection of subtle defects on the surface of photovoltaic cells remains challenging. As shown in Figure 1, Dynamic R-CNN [9] performs poorly in the localization of subtle defects, with detection regions appearing fragmented and discontinuous; YoLov10 [10] is prone to missing small defects, resulting in incomplete detection and DETR [11] often misclassifies regions unrelated to actual defects as defects.

To address the above issues, we propose a novel surface defect detector based on subtle defect enhancement and background suppression, which incorporates two key innovations: Subtle Feature Refinement Module (SFRM) and Background Noise Suppression Block (BNSB). Specifically, SFRM partitions spatial features and rearranges them into the channel dimension, combined with a channel attention mechanism, enhancing the representation of subtle defects and preserving their complete information. BNSB adopts a parallel dual-path structure to effectively suppress background noise interference: on the one hand, the first path incorporates the BAM to adaptively weaken irrelevant background information; on the other hand, the second path preserves the integrity of input features through a residual connection to prevent the loss of critical details. In summary, the main contributions of this paper are as follows:

- We propose a plug-and-play SFRM module that preserves subtle defect features by rearranging spatial information and enhances key feature representation through channel attention.
- We propose a dual-path BNSB, the first path uses BAM to suppress noise and highlight key features, and the second path employs residual structure to preserve the original features, thereby reducing background interference and enhancing defect saliency.
- Based on SFRM and BNSB, a novel detector PSDD is proposed to achieve efficient and accurate detection of PV cell defects by enhancing subtle features and suppressing noise.
- Extensive tests show that PSDD outperforms other advanced methods, achieving the best $mAP_{50}$ of 93.6% in the PVEL-AD datasets.

This paper is organized as follows. Section 2 reviews the progress of related research. Section 3 provides a detailed introduction to the proposed method. Section 4 presents the experimental results. Section 5 concludes the paper.

## 2. Related Work

### 2.1. Attention Mechanism

The attention mechanism, inspired by the selective focus of the human visual system, adaptively recalibrates feature representations by assigning higher weights to informative signals while suppressing redundant or noisy information. This dynamic weighting enhances the model’s ability to capture salient patterns and contextual dependencies, improving both discriminative power and generalization capability. The advantages of the attention mechanism include stronger feature representation, the ability to capture long-range dependencies, and improved robustness against irrelevant information. However, attention mechanisms also introduce higher computational and memory costs, increase network complexity, and may require large or high-quality datasets to achieve optimal performance. Attention mechanisms can be categorized into three types based on their operational dimensions: channel attention, spatial attention, and self-attention. The channel attention mechanism adaptively assigns weights to each channel, enhancing the representation of defect-related features while suppressing redundant information and background noise. Hu et al. [12] proposed the Squeeze-and-Excitation (SE) network, which extracts global contextual information through global average pooling and adaptively recalibrates channel-wise feature responses, thereby enhancing the network’s focus on informative channels while suppressing irrelevant features. The spatial attention mechanism adaptively adjusts the weights of different positions in the feature map based on global or local spatial information, highlighting salient features in key regions while suppressing interference from non-critical areas. Jongchan Park et al. [13] proposed the bottleneck attention module, which generates attention maps along the spatial dimension to reinforce the representation of salient regions, enabling more precise detection of defect areas. The self-attention mechanism dynamically weights and fuses input features based on the correlations between elements, thereby capturing global long-range dependencies and enhancing feature representation. Chen et al. [14] introduced a lightweight self-attention mechanism within the DETR framework to achieve real-time detection of surface defects in crystalline silicon photovoltaic cells, balancing global modeling capability with inference efficiency. Their method effectively improves the accuracy of defect identification.

### 2.2. Photovoltaic Cell Defect Detection

Deep learning-based methods have become the mainstream approach for PV cell defect detection due to their ability to automatically learn discriminative features from raw images. These methods can be broadly categorized into single-stage, two-stage, and Transformer-based approaches, based on differences in detection processes and model architectures. Single-stage detection methods offer high inference speed and are suitable for real-time applications, but their accuracy may be limited for small or densely packed defects. Fioresi et al. [15] proposed a detection framework based on an improved YOLOX [16] model, which can efficiently identify and localize defects even with a limited number of training samples. Two-stage detection methods provide higher accuracy and better handling of small or overlapping defects, but their multi-step processing increases model complexity and slows inference. Su et al. [17] proposed a complementary attention network that sequentially combines channel and spatial attention to suppress background noise and enhance defect features. Transformer-based detection methods excel at capturing complex and multi-scale defects but require high computational resources, limiting real-time applicability. However, they typically require higher computational resources, making them more suitable for applications that prioritize detection accuracy over real-time performance. Lang et al. [18] proposed a YOLO-based PV defect detection method that integrates attention mechanisms and Transformer modules, enhancing detection accuracy by effectively capturing and fusing spatial and semantic features.

Although the above methods for detecting PV cell defects have achieved certain results, their applicability remains limited due to insufficient consideration of the characteristics of subtle defects and the impact of complex background noise. To more effectively capture the characteristics of PV cell surface defects and enhance detection accuracy, we design a plug-and-play SFRM module, which integrates a spatial-to-channel mapping strategy with a channel attention mechanism to effectively mitigate the loss of subtle defect features during the downsampling process. Meanwhile, we propose the BNSB module with a dual-path fusion strategy: the main path incorporates the BAM module to suppress interference from complex background noise, while the auxiliary path adopts a residual connection structure to preserve original feature information. On this basis, we propose the PSDD method, whose overall framework is mainly supported by both SFRM and BNSB. The detailed implementation is presented in Section 3.

## 3. Method

### 3.1. Overall Architecture

**Figure 2:**
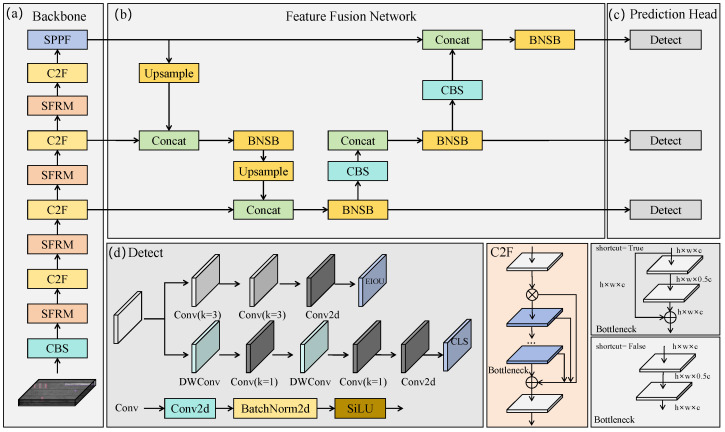
Overall architecture of our PSDD, which consists of the backbone network, feature fusion stage, and prediction head.

Figure 2 illustrates the overall architecture of our PSDD, which consists of three main components: the Backbone, the Feature Fusion Network (FFN), and the Prediction Head. We adopt Darknet53 [19] as the backbone to extract multiscale features ${\left\{f_{i}\right\}}_{i=1}^{3}$ from the input image $x\in R^{H\times W\times C}$ for subsequent processing, due to its strong feature extraction capability and low computational cost. To enhance the backbone’s ability to capture subtle defect patterns, we integrate a set of plug-and-play Subtle Feature Refinement Modules (SFRMs) into selected backbone layers, as shown in Figure 2a. We append the lightweight Spatial Pyramid Pooling Fast (SPPF) module at the end of the backbone to efficiently aggregate multi-scale spatial information and enhance global feature representation. FFN employs a Bidirectional Feature Pyramid strategy to integrate and refine multiscale features ${\left\{f_{i}\right\}}_{i=1}^{3}$. We further introduce the Background Noise Suppression Block (BNSB) in the feature fusion stage to suppress background interference and enhance the feature representation of foreground defects. For the final prediction stage, we adopt a decoupled head design that handles classification and regression tasks separately to improve detection accuracy and robustness. The prediction branch consists of two convolutional layers and one Conv2D layer, while the classification branch employs two separable convolutional layers in depth and one Conv2D layer [20], achieving precise defect localization while effectively reducing the number of parameters and computational cost. In this paper, the main innovative components include SFRM and BNSB, which are detailed in Section 3.2 and Section 3.3, respectively.

### 3.2. Subtle Feature Refinement Module (SFRM)

**Figure 3:**
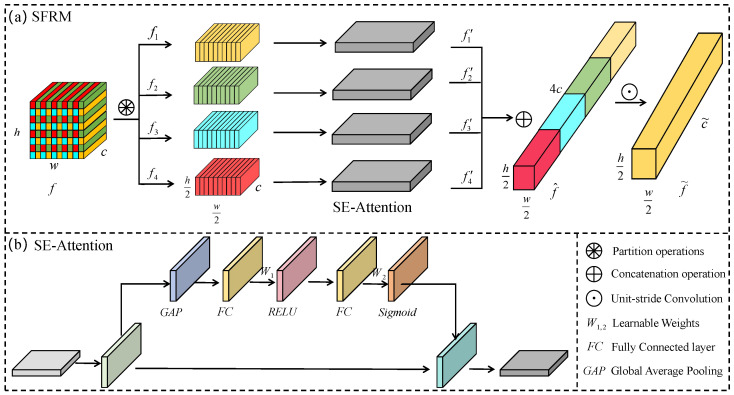
Architecture of the Subtle Feature Refinement Module.

Convolutional units are prone to losing fine-grained defect information as a result of strided convolutions and pooling operations. To mitigate this issue, we propose a plug-and-play SFRM that enhances the representation of subtle defect features. Figure 3a shows the architecture of the SFRM.

SFRM partitions the input feature $f\in R^{h\times w\times c}$ into four spatial subsets of equal size:

(1) $$\begin{array}{cc} f_{1} & =f[:,0::2,0::2,:]\\ f_{2} & =f[:,1::2,0::2,:]\\ f_{3} & =f[:,0::2,1::2,:]\\ f_{4} & =f[:,1::2,1::2,:] \end{array}$$

Each subset is represented as $f_{i}\in R^{\frac{h}{2}\times \frac{w}{2}\times c}$, achieving a twofold downsampling in spatial dimensions while fully preserving the original channel information. However, spatial division may also inadvertently retain background noise or irrelevant signals, thereby impacting the effectiveness of subsequent feature fusion. To mitigate this issue, an SE attention mechanism [12] is incorporated into SFRM (Figure 3b), which allows adaptive channel weighting to emphasize defect-relevant features. Formally,

(2) $$f_{i}^{\prime }=\sigma \left(W_{2}\;\delta \left(W_{1}\cdot \mathrm{GAP}\left(f_{i}\right)\right)\right)⊗f_{i},\;i\in \left\{1,2,3,4\right\}$$

The refined sub-features ${\left\{f_{i}^{\prime }\right\}}_{i=1}^{4}$ are concatenated along the channel axis to form a unified representation:

(3) $$\hat{f}=\mathrm{Concat}\left(f_{1}^{\prime },f_{2}^{\prime },f_{3}^{\prime },f_{4}^{\prime }\right)$$

Therefore, $\hat{f}\in R^{\frac{h}{2}\times \frac{w}{2}\times 4c}$ retains half the spatial resolution of the original input *f* while expanding the channel dimension fourfold to integrate complementary information from different subregions.

$\hat{f}$ is finally passed through a unit-stride convolutional layer with a kernel size of $\tilde{c}\times \tilde{c}$ to preserve subtle defect information:

(4) $$\tilde{f}={\mathrm{Conv}}_{1\times 1}^{\tilde{c}}\left(\hat{f}\right)$$

In this way, $\tilde{f}$ obtained by the SFRM preserves subtle defect information and thus may enhance the model’s discriminative capacity.

### 3.3. Background Noise Suppression Block (BNSB)

**Figure 4:**
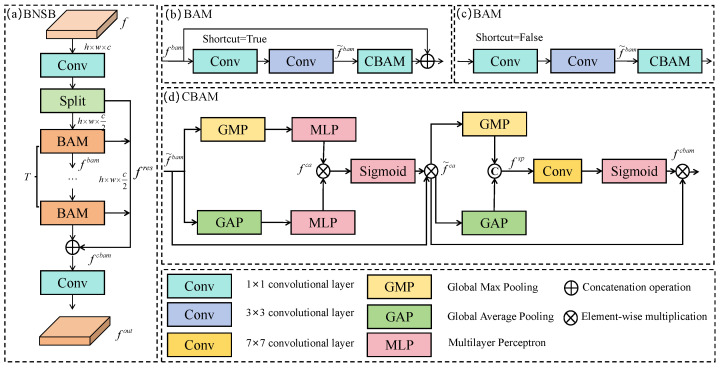
Structure of the background noise suppression block.

Features at different scales from the backbone network often contain noisy information. During the fusion process, background noise interference may be further amplified, which can obscure true defect features and reduce the discriminative capacity of the model. We design a novel BNSB module to alleviate noise interference using a dual path optimization strategy, as shown in Figure 4a.

Let $f\in R^{h\times w\times C}$ be the input feature obtained by fusing features from different levels, as illustrated in Figure 2. After reducing *f*’s channel dimension to *c* via a 1×1 convolution, BNSB splits it into two subsets, i.e., $f^{\mathrm{bam}}\in R^{h\times w\times \frac{c}{2}}$ and $f^{\mathrm{res}}\in R^{h\times w\times \frac{c}{2}}$. Formally,

(5) $$f^{\mathrm{bam}},f^{\mathrm{res}}=\mathrm{Split}\left(\mathrm{Conv}\left(f\right)\right)$$

$f^{\mathrm{bam}}$ is fed into the background noise suppression path, which consists of a sequence of background-aware modules (BAM) designed to suppress background noise.

(6) $$f_{i}=\left\{\begin{array}{c} \mathrm{BAM}(f^{\mathrm{bam}}),\;\mathrm{if}\;i=1\\ \mathrm{BAM}\left(f_{i-1}\right),\;\;\mathrm{if}\;i\in \left\{2,3,\ldots ,T\right\} \end{array}\right.$$

where *T*, defaulting to 4, denotes the number of BAMs. $f^{\mathrm{res}}$ is used as the residual path to retain detailed spatial information and complement the background suppressed features, followed by a 1×1 convolution to restore the dimension of the channel. Formally,

(7) $$f^{\mathrm{out}}=\mathrm{Conv}\left({⊕}_{i=1}^{T}f_{i}^{\mathrm{bam}}⊕f^{\mathrm{res}}\right)$$

Figure 4b,c shows the details of the proposed BAM with and without shortcut connections, respectively. BAM employs a bottleneck structure that compresses the channel dimension of $f^{\mathrm{bam}}$ using a 1×1 convolution, followed by a 3×3 convolution to extract local spatial features of defect regions, enhancing the model’s ability to perceive background noise. Formally,

(8) $${\tilde{f}}^{\mathrm{bam}}={\mathrm{Conv}}_{3\times 3}\left({\mathrm{Conv}}_{1\times 1}\left(f^{\mathrm{bam}}\right)\right)$$

The Convolutional Block Attention Module (CBAM) is applied to suppress background noise and enhance feature representation through channel and spatial attention mechanisms. Specifically, CBAM sequentially combines channel attention and spatial attention. ${\tilde{f}}^{\mathrm{bam}}$ undergoes channel attention, where both GAP and GMP are applied to extract two complementary pooled features. These features are then passed through MLP, followed by element-wise multiplication and sigmoid activation to generate channel attention $f^{\mathrm{ca}}$. Formally,

(9) $$f^{\mathrm{ca}}=\sigma \left(\mathrm{MLP}\left(\mathrm{GMP}\left({\tilde{f}}^{\mathrm{bam}}\right)⊗\mathrm{GAP}\left({\tilde{f}}^{\mathrm{bam}}\right)\right)\right)$$

The output $f^{\mathrm{ca}}$ is then element-wise multiplied with ${\tilde{f}}^{\mathrm{bam}}$ to generate the channel-refined feature ${\tilde{f}}^{\mathrm{ca}}$:

(10) $${\tilde{f}}^{\mathrm{ca}}={\tilde{f}}^{\mathrm{bam}}⊗f^{\mathrm{ca}}$$

CBAM applies spatial attention to ${\tilde{f}}^{\mathrm{ca}}$. Specifically, GAP and GMP perform along the spatial dimensions in ${\tilde{f}}^{\mathrm{ca}}$ to extract complementary spatial context information. The pooled results are then concatenated to form the spatial attention input $f^{\mathrm{sp}}$, formulated as follows:

(11) $$f^{\mathrm{sp}}=\mathrm{concat}\left(\mathrm{GAP}\left({\tilde{f}}^{\mathrm{ca}}\right),\mathrm{GMP}\left({\tilde{f}}^{\mathrm{ca}}\right)\right)$$

$f^{\mathrm{sp}}$ is processed by a 7×7 convolution to extract spatial contextual information, followed by a sigmoid activation function to generate spatial attention weights. These weights are then element-wise multiplied with $f^{\mathrm{ca}}$, resulting in the final output feature of BAM, denoted as $f^{\mathrm{cbam}}$.

(12) $$f^{\mathrm{cbam}}=\sigma \left({\mathrm{Conv}}_{7\times 7}\left(f^{\mathrm{sp}}\right)\right)⊗{\tilde{f}}^{\mathrm{ca}}$$

### 3.4. Loss Function

We use Enhanced IoU (EIoU) [21] loss to improve boundary sensitivity and precisely localize subtle defects by leveraging the geometric structure. Formally,

(13) $$\begin{array}{cc} L_{\mathrm{EIoU}} & =L_{\mathrm{IoU}}+L_{\mathrm{dls}}+L_{\mathrm{asp}}\\ L_{\mathrm{IoU}} & =1-\mathrm{IoU}\\ L_{\mathrm{dls}} & =\frac{\rho^{2}\left(b,b^{\mathrm{gt}}\right)}{{\left(w^{c}\right)}^{2}}\\ L_{\mathrm{asp}} & =\frac{\rho^{2}\left(w,w^{\mathrm{gt}}\right)}{{\left(w^{c}\right)}^{2}}+\frac{\rho^{2}\left(h,h^{\mathrm{gt}}\right)}{{\left(h^{c}\right)}^{2}} \end{array}$$

where IoU (Intersection over Union) [21] measures the overlap between predicted and ground-truth boxes and reflects the localization accuracy. Distance loss $L_{\mathrm{dls}}$ normalizes center point deviation to improve positioning precision, while the aspect ratio loss $L_{\mathrm{asp}}$ penalizes size mismatches to maintain shape consistency and enhance defect detection performance.

## 4. Experiments

### 4.1. Experimental Setup

#### 4.1.1. Datasets

We conduct experiments on the publicly available PVEL-AD dataset [22] to evaluate the performance of our PSDD. PVEL-AD, jointly released by Hebei University of Technology and Beihang University, is a high-quality near-infrared EL dataset comprising 36,543 images with diverse internal defects and heterogeneous backgrounds across 12 defect types. Due to the extremely limited number of samples for Printing_Error, Corner, Fragment, and Scratch defect types, we constructed a representative subset consisting of 3524 images. This subset contains eight common defect types: Black_Core (Bc), Crack (Cr), Finger (Fi), Horizontal_Dislocation (Hd), Short_Circuit (Sci), Star_Crack (Scr), Thick_Line (Tl), and Vertical_Dislocation (Vd). Based on their spatial distribution and visual scale, these defects are grouped into large-scale (Bc, Hd, Vd, Sci) and subtle (Cr, Scr, Fi, Tl) types. This dataset is divided into training, validation, and test sets in a ratio of 7:1:2 to ensure the rationality of model training, parameter tuning, and performance evaluation.

#### 4.1.2. Implementing Details

Our PSDD is implemented using the PyTorch(1.13.1) framework and is trained on an NVIDIA A100 GPU with 80 GB of memory. During training and evaluation, all images are uniformly resized to a resolution of 640 × 640 pixels. Stochastic Gradient Descent (SGD) [23] is employed as the optimizer, with a learning rate of 0.01, a momentum factor of 0.937, and a batch size of 32. The maximum number of training epochs is 200.

### 4.2. Evaluation Metrics

We use $mAP_{50}$ and $mAP_{50:95}$ as key metrics to evaluate detection performance. $mAP_{50}$ represents the average precision across all *N* categories at a fixed IoU threshold of 0.50, defined as follows:

(14) $$mAP_{50}=\frac{1}{N}\sum_{i=1}^{N}AP_{i}^{IoU=0.50}$$

$mAP_{50:95}$ is computed as the mean AP across *T* IoU thresholds from 0.50 to 0.95 with an interval of 0.05, defined as follows:

(15) $$mAP_{50:95}=\frac{1}{T}\sum_{t=1}^{T}\left(\frac{1}{N}\sum_{i=1}^{N}AP_{i}^{IoU=\tau_{t}}\right)$$

where T=10 corresponds to the number of evaluated thresholds. $AP_{i}^{IoU=\tau_{t}}$ is the Average Precision calculated for category *i* at that threshold, defined as follows:

(16) $$AP=\int_{0}^{1}P\left(R\right)\;dR$$

where P(R) denotes the function that describes the relationship between precision (*P*) and recall (*R*), respectively.

### 4.3. Comparative Experiment

We conducted comparative experiments between our PSDD and eighteen advanced detectors on the PVEL-AD dataset to verify the effectiveness of PSDD. These detectors span three categories: single-stage detectors, including TOOD [24], YOLOv8 [25], YOLOv10 [10], YOLOv11 [20], YOLOX [16], and Mamba YOLO [26]; two-stage detectors, including Dynamic R-CNN [9], Faster R-CNN [27], Mask R-CNN [28], Cascade RPN [29], SSOD [30], and IOD [31] and transformer-based detectors, including DETR [11], Deformable DETR [32], Rt-DETR [33], Swin-Transformer [34], DINO [35], and Wave-ViT [36]. The comparative experiments cover key metrics, including ${\mathrm{mAP}}_{50}$, ${\mathrm{mAP}}_{50:95}$, precision, recall, model parameters, FLOPs, inference speed, and detection performance across all defect categories, with the overall performance and category-specific results summarized in Table 1.

As shown in Table 1, PSDD achieves 93.6%, 65.3%, and 90.8% on ${\mathrm{mAP}}_{50}$, ${\mathrm{mAP}}_{50:95}$, and *P*, respectively, ranking highest among all compared models. In terms of inference speed, PSDD processes a single image in only 10.0 ms, slightly faster than the single-stage detector Yolov11 [20] (12.0 ms). This advantage is primarily attributed to PSDD’s efficient design of feature extraction and detection heads, which enables low latency while maintaining high accuracy. In contrast, the two-stage detectors SSOD [30] and IOD [31] require 25.6 ms and 19.2 ms per image, respectively. This is mainly because SSOD suffers from efficiency bottlenecks in feature fusion and region proposal generation, while IOD has relatively limited capability in modeling fine-grained defects, resulting in lower overall inference speed and detection performance. In terms of model complexity, PSDD contains only 4.0M parameters and 13.8G FLOPs, achieving an excellent balance between detection accuracy and computational cost. Wave-ViT [36], due to its complex self-attention mechanisms, has a substantially larger model size, with 66.1M parameters and 96.4G FLOPs. In addition, PSDD also demonstrates outstanding performance across all defect categories, achieving the highest mAP in five categories (Bc, Cr, Scr, Fi, and Tl) at 98.8%, 84.7%, 87.0%, 90.4%, and 91.3%, respectively. Notably, its detection performance on subtle defects such as Cr, Scr, Fi, and Tl is particularly strong, further validating the model’s robustness in high-precision defect detection tasks. After approximately the 125th epoch, PSDD significantly outperforms all other models in $mAP_{50}$, demonstrating its strong learning capability and faster convergence.

**Figure 5:**
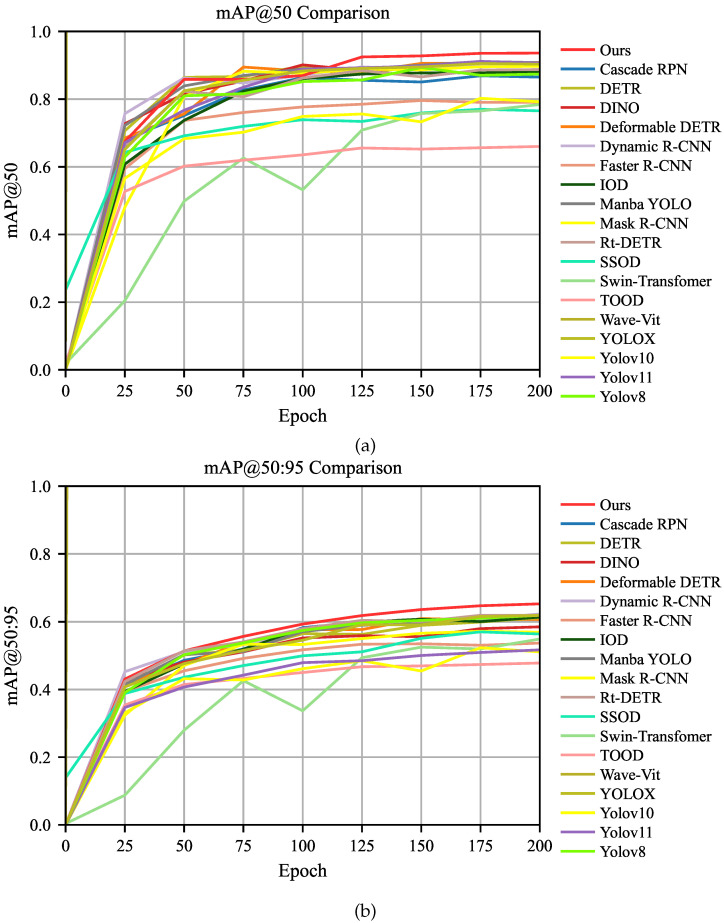
The mAP variation curves of PSDD compared to other models on the PVEL-AD dataset. (**a**,**b**) depict the trends of ${mAP}_{50}$ and ${mAP}_{50:95}$, respectively.

Figure 5 presents the changes in mAP metrics of various comparison models on the PVEL-AD dataset over training iterations. The red curve at the top of the figure represents PSDD. The results indicate that the performance of PSDD gradually improves with training iterations and ultimately reaches the optimal result.

Notably, after approximately the 125th epoch, PSDD significantly outperforms all other models in mAP50, demonstrating its strong learning capability and faster convergence. In addition, PSDD consistently maintains an advantage in the $mAP_{50:95}$ metric, indicating high sensitivity to capture defect details and stable recognition performance.

### 4.4. Ablation Experiments

To further analyze the contribution of each component, we progressively integrate the SFRM and BNSB modules into the baseline model and conduct ablation experiments. The results are reported in Table 2.

When the SFRM module is added to the baseline model alone, the model’s *mAP*50 increased by 2.5% (from 87.4% to 89.9%), with *P* and *R* improving by 1.6% and 6.4%, respectively. In particular, the most significant performance gains are observed in the Cr and Scr categories, increasing from 78.9% to 84.7% and from 73.2% to 81.2%, respectively. These results indicate that SFRM, by combining a spatial-to-channel mapping strategy with a channel attention mechanism during feature downsampling, assigns higher responses to subtle and critical defect regions, effectively preserving fine defect information and thereby enhancing the model’s capability to detect subtle defects.

When the BNSB module is integrated into the baseline model alone, the model’s $mAP_{50}$ increases by 4.7% (reaching 92.1%), with *P* and *R* improving by 3.6% and 3.8%, respectively. The most significant performance gain is observed in the Vd category, increasing from 73.0% to 96.8%, while notable improvements are also achieved in the Fi and Tl categories. This improvement is mainly attributed to BNSB, which introduces the BAM module in the main path to effectively suppress complex background noise, while retaining residual connections in the auxiliary path to preserve original feature information, thereby significantly enhancing the representation of defect features. This mechanism not only improves the model’s detection performance for categories with strong background interference but also optimizes the overall detection accuracy.

**Figure 6:**
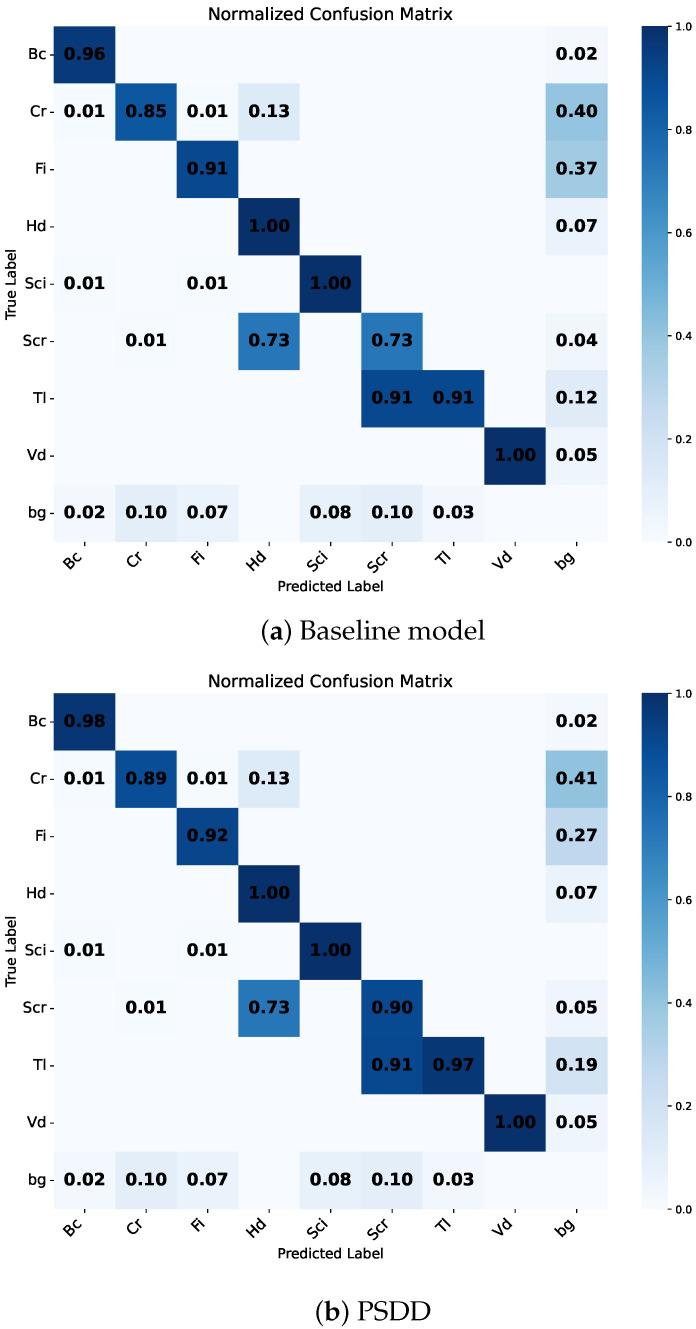
Confusion matrix comparison between the baseline and PSDD models. Labels represent a 9-class classification, including ‘bg’ for background (non-defective regions).

When the SFRM and BNSB modules are integrated simultaneously, the model achieves the best overall performance, with $mAP_{50}$, *P*, and *R* reaching 93.6%, 90.8%, and 89.2%, respectively. Furthermore, as shown in Figure 6, we compare the confusion matrix of the PSDD model (Figure 6b) with that of the baseline model (Figure 6a) to analyze their performance differences in the detection of various defect categories. Compared with the baseline model, PSDD improves the detection accuracy for the subtle defect categories Cr, Scr, and Tl by 0.04, 0.17, and 0.06, respectively. It demonstrates that our PSDD has superior capability in identifying subtle defects under complex backgrounds.

### 4.5. Visualization

**Figure 7:**
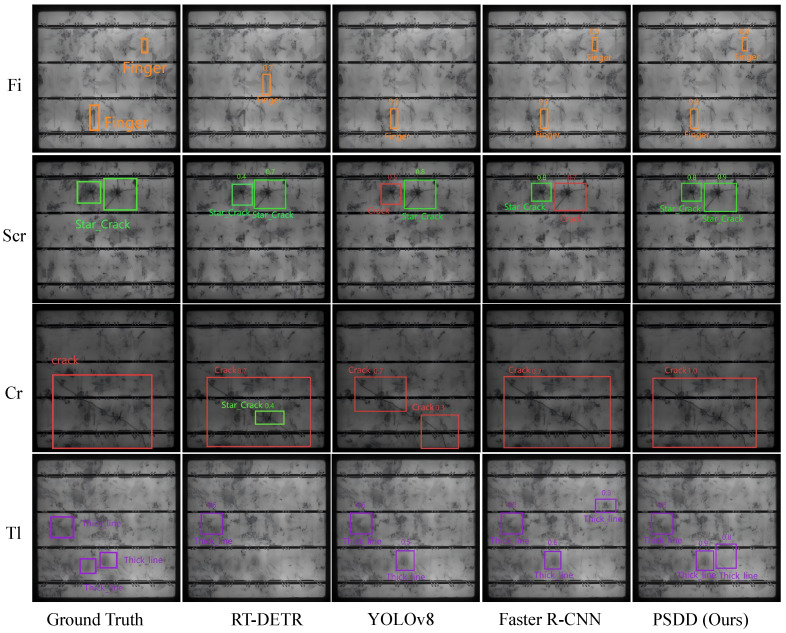
Visualization comparison of different models on PVEL-AD.

Figure 7 presents a visual comparison between the proposed PSDD method and several mainstream object detectors on the PVEL-AD dataset. As shown in Figure 7, PSDD is capable of precisely localizing defect regions while maintaining stable confidence distributions, achieving high consistency with the ground-truth defect areas and demonstrating superior detection accuracy and robustness. In contrast, RT-DETR [33] fails to fully recognize Fi and Tl defects and produces false detections in the Cr category; YOLOv8 [25] exhibits missed detections for Fi and Tl, fragments the Cr defect into two separate regions, and misclassifies Scr as Cr; Faster R-CNN [27] tends to confuse Scr with Cr and assigns relatively low confidence scores to Fi and Tl.

**Figure 8:**
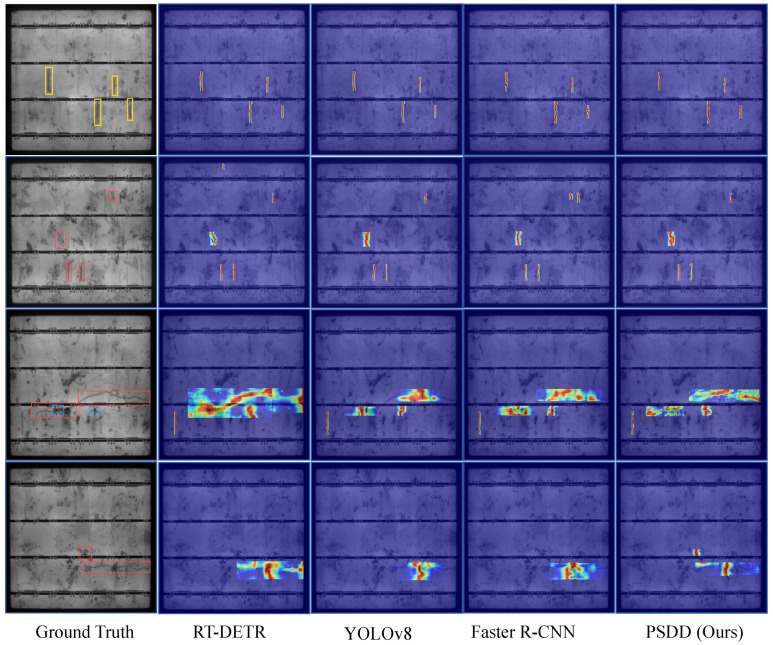
Heatmap visualization comparison of different models on the PVEL-AD dataset. The yellow boxes indicate finger defects, the red boxes indicate crack defects, and the blue boxes represent star-shaped crack defects.

We conducted Class Activation Mapping (CAM) visualization analysis for PSDD and several comparative models [37] to further evaluate PSDD’s capability in detecting subtle defects under complex backgrounds and to enhance its interpretability [37]. The results in Figure 8 indicate that PSDD can accurately focus on subtle defect regions and generate strong activations at critical locations. The detected regions align closely with the ground-truth defect areas, underscoring the model’s accuracy and reliability in fine-grained defect localization. In contrast, Rt-DETR [33] exhibits relatively broad activation regions, which weaken defect responses and increase the risk of false positives; YOLOv8 [25] shows insufficient feature representation for fine-grained defects, with dispersed activations failing to effectively cover defect areas; Faster R-CNN [27] produces some focused activations in certain discrete defect regions, but its overall response strength and spatial continuity are limited, constraining its ability to identify subtle defects in complex textured environments. In summary, PSDD demonstrates significant advantages in feature extraction and spatial localization, effectively improving the accuracy and robustness of subtle defect detection.

### 4.6. Generalization Experiments

To comprehensively assess the generalization performance of the proposed PSDD model, this study selects eleven representative object detection models for comparison, covering the mainstream detection architectures. Among them, the single-stage detection models include TOOD [24], YOLOv8 [25], YOLOv10 [38], YOLOv11 [20], YOLOX [16], and Mamba YOLO [26]; the two-stage detection model includes Faster R-CNN [27] and the Transformer-based detection models include Deformable DETR [32], Rt-DETR [33], and Swin-Transformer [34]. To systematically compare the performance of these models, their overall performance was quantified using $mAP_{50}$, *P*, and *R*, followed by a detailed evaluation of detection accuracy across different defect categories. All experiments are carried out on the NEU-Det dataset [39], a widely used benchmark for steel surface defect detection released by Northeastern University. The NEU-Det dataset consists of 1800 grayscale images with a resolution of 200 × 200 pixels, evenly distributed among six typical defect types. Among them, Crazing (Cr), Inclusion (In), and Pitted Surface (Ps) are fine-grained defects characterized by small sizes and low contrast, whereas Patches (Pa), Rolled-in Scale (Rs) and Scratches (Sc) are large-scale defects with clear contours.

Table 3 presents a comparative evaluation of PSDD against other representative object detection models on the NEU-Det dataset. PSDD achieves the best overall performance, with a $mAP_{50}$ of 80.9%, *P* of 81.4%, and *R* of 80.6%. Across different defect categories, PSDD attains high detection accuracy for Cr (52.4%), In (84.1%), Pa (93.4%), Ps (90.6%), and Sc (96.4%). In particular, it demonstrates a clear advantage in detecting low-contrast and subtle defects (Cr and In), highlighting its enhanced capability to identify subtle defects under complex backgrounds.

## 5. Conclusions

This paper proposes a novel photovoltaic cell surface defect detector (PSDD), designed to improve detection performance under complex background conditions. Compared with existing methods, including traditional CNN-based detectors and attention-guided architectures, PSDD demonstrates superior capability in enhancing subtle defect features and effectively suppressing background noise. This improvement is primarily attributed to the Subtle Feature Refinement Module (SFRM), which captures fine-grained defect information through spatial feature splitting and channel attention, and the Background Noise Suppression Block (BNSB), which employs a dual-path fusion strategy to balance noise suppression and information retention. Experimental results in the public PVEL-AD dataset further validate the advantages of PSDD. In addition to achieving a $mAP_{50}$ of 93.6%, the results demonstrate the effectiveness of SFRM in extracting subtle defect features and the role of BNSB in distinguishing foreground defects under complex backgrounds. Meanwhile, we note that PSDD may face challenges in detecting extremely small or low-contrast defects, and the added modules slightly increase computational complexity. Therefore, future work will explore optimization strategies for extremely small and low-contrast defects and attempt to refine module designs to reduce computational overhead, thereby enhancing practicality and detection performance. At the same time, we plan to conduct evaluations on more diverse photovoltaic defect datasets to assess the generalization capability of PSDD and comprehensively examine its robustness under varying environmental and imaging conditions.

## Figures and Tables

**Table 1:** Performance comparison of multiple models on the PVEL-AD dataset and mAP (%) results across all defect categories.

Model	${\mathrm{mAP}}_{50}$	${\mathrm{mAP}}_{50:95}$	*P*	*R*	Params (M)	FLOPs (G)	Speed (ms)	Bc	Cr	Scr	Fi	Tl	Sci	Hd	Vd
Single-stage Detectors															
TOOD [24]	66.0	47.9	61.3	62.7	3.7	17.8	**5.9**	98.0	75.2	78.1	89.3	88.2	99.2	–	–
Yolov8 [25]	87.4	61.9	90.3	81.5	**3.5**	8.1	9.0	97.0	78.9	73.2	88.9	89.7	99.5	98.8	73.0
Yolov10 [10]	79.2	51.1	78.4	72.6	6.5	16.6	17.4	98.6	77.4	79.2	90.6	91.2	99.5	98.5	89.4
Yolov11 [20]	90.6	51.7	78.9	72.9	8.0	14.5	12.0	98.3	83.8	83.7	89.7	90.8	99.5	98.5	74.4
YoloX [16]	90.3	62.2	85.7	87.1	9.0	**7.0**	20.0	98.4	80.6	81.8	89.8	89.8	99.5	98.6	83.9
Mamba YOLO [26]	91.6	62.1	87.9	85.5	5.8	13.2	14.4	98.8	80.7	80.7	89.9	90.8	99.4	96.2	96.9
Two-stage Detectors															
Dynamic R-CNN [9]	89.8	62.3	83.2	88.8	45.0	46.3	24.0	98.8	76.3	68.5	90.3	82.4	**99.7**	81.6	–
Faster R-CNN [27]	79.7	54.0	77.0	75.9	23.3	26.4	22.0	97.5	75.2	82.7	89.3	89.4	99.2	98.5	–
Mask R-CNN [28]	90.1	57.6	85.5	82.5	30.0	33.3	26.0	98.1	77.7	83.9	88.7	88.4	98.0	96.7	89.4
Cascade RPN [29]	87.8	61.9	87.6	79.5	47.3	136.6	35.5	98.8	79.6	82.2	89.7	88.9	99.5	96.9	67.9
SSOD [30]	77.3	52.4	76.9	71.3	26.4	19.2	25.6	98.0	80.7	80.8	83.6	88.2	98.5	89.3	–
IOD [31]	87.8	61.8	89.2	80.4	9.0	14.5	19.2	98.4	79.5	82.0	87.9	88.9	98.4	97.2	70.2
Transformer-based Detectors															
DETR [11]	90.4	61.2	78.9	**89.7**	41.6	60.5	27.0	95.8	80.1	71.8	87.4	90.0	99.2	86.8	**99.5**
Deformable DETR [32]	91.2	61.9	85.4	87.0	39.9	52.4	24.5	97.7	80.96	83.8	89.7	90.0	99.4	98.5	90.8
Rt-DETR [33]	89.1	63.0	89.3	78.9	19.9	57.0	20.7	98.3	82.1	78.9	89.9	90.3	98.3	97.5	77.4
Swin-Transformer [34]	78.4	54.6	71.3	87.1	54.6	136.0	33.7	89.9	60.0	42.1	88.9	74.8	99.6	**99.6**	69.7
DINO [35]	90.4	59.0	83.2	87.2	59.3	123.0	32.0	98.7	80.1	86.8	89.9	91.1	99.5	89.3	86.7
Wave-ViT [36]	90.6	61.8	85.9	88.3	66.1	96.4	12.4	98.6	78.2	81.0	89.9	91.1	99.5	96.3	90.6
**PSDD (Ours)**	**93.6**	**65.3**	**90.8**	89.2	4.0	13.8	10.0	**98.8**	**84.7**	**87.0**	**90.4**	**91.3**	99.5	99.3	99.4

The best results are highlighted in bold, and ‘–’ indicates unavailable results.

**Table 2:** Results of ablation experiments for each module with 8 defect types.

Module			${\mathrm{mAP}}_{50}$	${\mathrm{mAP}}_{50:95}$	*P*	*R*	Bc	Cr	Scr	Fi	Tl	Sci	Hd	Vd
Baseline	SFRM	BNSB	(%)	(%)	(%)	(%)	(%)	(%)	(%)	(%)	(%)	(%)	(%)	(%)
✓	–	–	87.4	61.9	83.6	81.5	97.0	78.9	73.2	77.2	89.7	99.5	98.8	73.0
✓	✓	–	89.9	62.7	85.2	87.9	98.3	84.7	81.2	89.6	89.4	99.3	99.4	77.5
✓	–	✓	92.1	64.5	87.2	85.3	98.2	80.8	81.0	90.9	90.4	99.5	99.4	96.8
✓	✓	✓	93.6	65.3	90.8	89.2	98.8	84.7	87.0	90.4	91.3	99.5	99.3	99.4

✓ indicates that the corresponding module is included; “–” means the module is not included.

**Table 3:** Comparison results of different models on the NEU-Det dataset.

Model	${\mathrm{mAP}}_{50}$	*P*	*R*	Cr	In	Pa	Ps	Rs	Sc
TOOD [24]	72.4	75.7	68.1	42.8	71.5	87.7	80.9	59.9	91.9
Yolov8 [25]	77.3	75.4	70.8	49.7	77.9	90.1	88.0	63.7	94.7
Yolov10 [38]	74.2	68.3	69.7	40.2	74.3	91.9	83.3	61.4	94.4
Yolov11 [20]	79.4	79.1	71.7	51.0	79.5	92.3	87.3	**70.9 **	95.2
YoloX [16]	74.5	70.4	69.7	37.8	79.3	90.3	86.0	60.6	92.5
Faster R-CNN [27]	70.9	67.4	66.7	36.2	74.2	89.1	77.9	57.0	90.9
Rt-DETR [33]	70.6	69.3	68.1	33.3	73.5	88.0	81.7	55.4	91.6
Deformable DETR [32]	71.1	70.3	67.2	30.5	76.3	89.1	82.7	56.2	91.6
Swin-Transformer [34]	70.3	69.9	65.3	31.8	74.2	88.0	79.3	57.2	90.9
Mamba YOLO [26]	69.6	72.3	63.7	30.7	73.8	88.7	76.5	58.0	90.1
PSDD (Ours)	**80.9**	**81.4**	**80.6**	**52.4**	**84.1**	**93.4**	**90.6**	68.3	**96.4**

Note: Best results are shown in bold.

## Data Availability

Our code are available at https://github.com/tm924222/PSDD (accessed on 27 August 2025).
